# Exploring the role of ATP-binding cassette transporters in tomato (*Solanum lycopersicum*) under cadmium stress through genome-wide and transcriptomic analysis

**DOI:** 10.3389/fpls.2025.1536178

**Published:** 2025-03-18

**Authors:** Syed Salman Hashmi, Lubna, Saqib Bilal, Rahmatullah Jan, Saleem Asif, Ashraf M. M. Abdelbacki, Kyung-Min Kim, Ahmed Al-Harrasi, Sajjad Asaf

**Affiliations:** ^1^ Natural and Medical Sciences Research Center, University of Nizwa, Nizwa, Oman; ^2^ Coastal Agriculture Research Institute, Kyungpook National University, Daegu, Republic of Korea; ^3^ Department of Applied Biosciences, Kyungpook National University, Daegu, Republic of Korea; ^4^ Deanship of Skills Development, King Saud University, Riyadh, Saudi Arabia

**Keywords:** *Solanum lycopersicum*, heavy metals, ABC transporters, phylogenetic analysis, gene expression

## Abstract

ATP-binding cassette (ABC) transporters are integral membrane proteins involved in the active transport of various substrates, including heavy metals, across cellular membrane. In this study, we performed a genome-wide analysis and explored the expression profiles of ABC transporter genes in *Solanum lycopersicum* to identify their role in cadmium (Cd) stress tolerance. Several techniques were employed to determine the regulatory role of ABC transporters. A total of 154 ABC transporter genes were identified in the genome of *S. lycopersicum*, located on all 12 chromosomes. Comparative phylogenetic analysis between *S. lycopersicum* and *Arabidopsis thaliana* revealed several orthologous gene pairs, which were duly supported by the structural analysis of the genes by studying the exon-intron pattern and motif analysis. Collinearity analysis revealed multiple gene duplication events owing to intra-chromosomal and inter-chromosomal mutations. The cis-regulatory analysis identified several hormone-responsive elements suggesting that ABCs are actively involved in transporting hormones like ABA, SA, MeJA, auxin, and gibberellin. These hormones are known to combat a number of stress conditions, hence validating the role of ABCs in Cd stress. Under Cd stress, expression profiling demonstrated that several *SlABCs* exhibit significant transcriptional changes, indicating their involvement in Cd transport, sequestration, and detoxification mechanisms. Specific genes, including Groups 3 and 5 members, were upregulated under Cd exposure, suggesting their functional roles in mitigating Cd toxicity. The study revealed differential expressions of various *SlABC* genes encoding ATP binding cassette transporters, including the upregulation of several genes like *Solyc08g067620.2*, *Solyc08g067610.3*, *Solyc12g019640.2*, *Solyc06g036240.2*, and *Solyc05g053610.2* in response to different concentrations of Cd. This study comprehensively explains the ABC transporter gene family in *S. lycopersicum*, emphasizing their critical roles in Cd stress tolerance. This study could prove useful in combating Cd stress not only in *S. lycopersicum* but also in other fleshy fruit plants; however, further advanced studies on specific pathways that lead to differential expression of the ABC genes are required to understand the mechanism behind tolerance to heavy metals fully.

## Introduction

*S. lycopersicum*, a member of the Solanaceae family, commonly known as the tomato plant, is widely cultivated for its edible product, i.e., tomato. It is among the most cultivated vegetables across the globe, with estimated production of 187 million tons (Arshad et al., 2024). It is widely used for making a variety of dishes and sauces. Lycopene, phenolics, flavonoids, carotenoids, gallic acid, quercetin, vitamin A, vitamin B, and vitamin C make *S. lycopersicum* an important medicinal plant. These bioactive compounds have potential anti-cancer, anti-inflammatory, radio-protective, antibacterial, and antifungal activity. *S. lycopersicum* also possess the potential to alleviate symptoms of metabolic syndromes like diabetes, hypertension, diabetes-induced testicular injuries, and hypercholesterolemia (Kumar et al., 2021; Mallick, 2021; Collins et al., 2022). In nature, plants have to cope with numerous environmental conditions that induce stress. The stress may be induced by living organisms, i.e., viruses, fungi, bacteria, insects, and other pathogens (biotic), or their non-living environment, i.e., drought, salinity, floods, nutrient deficiency, heavy metals (HMs), and extreme temperatures (abiotic). Since plants are sessile, escaping biotic and abiotic stress is not an option, and prolonged exposure may have a detrimental impact on plant growth and development (Iqbal et al., 2021; Zhang et al., 2023).

The presence of HMs in large quantities in the soil is a matter of great concern globally (Kaur et al., 2021). Any metal with an atomic density higher than 4 g.cm^-3^ is considered a heavy metal e,g. nickel (Ni), lead (Pb), copper (Cu), mercury (Hg), zinc (Zn), aluminum (Al), chromium (Cr), and cadmium (Cd). The levels of HM pollution in our environment is rising at an alarming rate due to natural factors (acid rain and volcanic eruptions etc.) and human activities (mining, agriculture and industries) (Feng et al., 2021). HMs pose a long-term threat to the natural environment and human survival because of their non-degradable nature, long-term presence, and invisibility in the environment (Suman et al., 2018). Within cells, HMs affect photosynthesis, respiration, mineral nutrition, enzyme reactions, and many other physiological processes (Pourrut et al., 2011). The excessive accumulation of HMs in plant tissues can reduce root length, plant biomass, seed germination, and chlorophyll synthesis (Shahid et al., 2014). The inhibition of root growth, especially at the tip, is susceptible to certain HMs such as Al, Cu, Cd, and Hg (Gupta et al., 2014). The effects of HMs on plants are multifaceted, mainly including plant growth, plant dry weight, net photosynthesis, and stomatal conductance etc (Xiong et al., 2019). Plants, when exposed to HMs, result in metabolic cascades involving a plethora of genes. This results in the production of biochemicals that help plants endure HM stress (Khan et al., 2023a). These biochemicals are readily transported by membrane transport proteins, including transmembrane proteins like ATP-binding cassette (ABC) transporters (Ofori et al., 2018).

The ABC transporter factor family is found in various plant species, with different numbers of genes identified in each species. For example, *Arabidopsis thaliana* has 127 ABC genes, *Oryza sativa L.* has 123, and *Hordeum vulgare* has 131 (Guo et al., 2022). Initially, these transporters were identified for their role in cell detoxification by removing harmful chemicals from the cytoplasm. However, further research has demonstrated that ABC transporters are involved in a wide range of biological functions in plants, primarily situated in cellular membranes (Do et al., 2018). These genes have diverse functions, including transporting hormones, lipids, metals, pathogens, antibiotics, secondary metabolites, and other substances. They play a vital role in plant-pathogen interactions and ion channel regulation (Guo et al., 2022). Apart from this, it also affects seed dormancy, grain shape and size regulation, accumulation of anti-nutritional factors in seeds, transport of abscisic acid (ABA) and other phytohormones, growth and differentiation, nutrient uptake, redox balance, and endosperm weakening (Kang et al., 2011; Do et al., 2018).

ABC also plays a critical role in a variety of stress conditions. In a study by An et al. (An et al., 2019), *Betula halophile*, a plant with the ability to survive extreme drought and salinity stress was reported to contain 15 diverse ABC transporter genes. The ABC family transporter *HMT1* plays a significant role in the uptake of HMs through chelation and their subsequent partitioning into the vacuole. Similarly, *AtABCC1* and *AtABCC2* are crucial for metal uptake and sequestration, facilitating the transport of heavy metal-phytochelatin complexes into the vacuole, especially for metals like arsenic [As(III)], Cd [Cd(II)], and mercury [Hg(II)] (Park et al., 2009; Yang and Murphy, 2009; Song et al., 2010). ABC transporters protect tissues from metal toxicity in plant roots, facilitate auxin transport, and contribute to suberin deposition. In leaves, these transporters play role in ABA import, cuticle formation, and ion transport (Do et al., 2018). ABC transporters are vital for protecting pollen from environmental stresses and ensuring proper fertilization and seed development (Luo et al., 2020). In the shoots, they are involved in auxin transport, transporting fatty acyl CoA to peroxisomes, forming the cuticle, and generating plastid lipids (Do et al., 2018). They also contribute to forming the exine, the outer pollen wall, and aid in accumulating minerals, lipids, and cutin in seeds (Kang et al., 2011). Additionally, tonoplastic ABC transporters such as *ABCB27* and *OsALS1* have been linked to Al tolerance. The role of *AtABCC1* and *AtABCC2* in the vacuolar sequestration of Cd was effectively demonstrated using a fluorescent probe sensitive to Cd (Song et al., 2014). Furthermore, *AtABCB25*, a mitochondrial ABC transporter involved in synthesizing iron-sulfur clusters, was upregulated in the roots of Cd-treated plants. Overexpression of *AtABCB25* was shown to enhance Cd resistance (Song et al., 2014). The findings strongly indicate that ABC transporters likely play a crucial role in regulating the absorption and movement of both vital nutrients and HMs within organism. This suggests that they could influence how much nutrients or HMs accumulate in tissues. ABCs potentially impact nutrient utilization on nutrient-poor soils and the ability of plants to detoxify HMs through phytoremediation.

A genome-wide analysis is the comprehensive identification of all genes of the respective family including their family members and organization of their information. This approach provides essential information, such as evolutionary history, diversity, and relationship among genes and proteins, which serve as useful fundamental resources for further investigations. Genome-wide analyses of ABC transporters in Arabidopsis (Sánchez-Fernández et al., 2001), rice (Garcia et al., 2004), maize (Pang et al., 2013), *Lotus japonicas* (Sugiyama et al., 2006), grape (Çakır and Kılıçkaya, 2013), pineapple (Chen et al., 2017), and *Hevea brasiliensis* (Zhiyi et al., 2015) have already been performed. Ofori et al (Ofori et al., 2018). carried out a genome-wide analysis of a tomato plant and identified 154 genes in its genome that encode for ABC proteins. The study also reports a phylogenetic analysis of these genes, providing valuable insights into the evolutionary history of ABC proteins found in *S. lycopersicum*. The study proved to be a gateway for further insights into ABC protein studies in *S. lycopersicum* and the family Solanaceae; however, to date, no data is available on the involvement and differential expression of the aforementioned genes in *S. lycopersicum* plants when exposed to HMs. Additionally, further focused research is needed to better understand how ABC transporters function in nutrient uptake, transport, and maintaining internal balance, as well as their role in detoxifying HMs. In this regard, we conducted an in-depth exploration of the ABC gene family in *S. lycopersicum* through a combination of in-silico analysis and experimental approaches. First, we performed a genome-wide analysis of the *S. lycopersicum* ABC genes and followed up with a greenhouse experiment to examine how these genes respond to varying concentrations of Cd. Additionally, we utilized RNA-Seq to analyze gene expression patterns and further validated our findings through qRT-PCR (Real-Time Quantitative Reverse Transcription Polymerase Chain Reaction). The goal of this research was to better understand the role of ABC genes in *S. lycopersicum’s* response to Cd stress, integrating both computational and experimental data for a comprehensive analysis.

## Materials and methods

### Growth condition under Cd stress

The current research was conducted on the Tomato plant (*S. lycopersicum* c v. Yegwang). Seeds were surface sterilized according to previously reported protocols (Khan et al., 2023b). The seeds were properly washed with distilled water (dH_2_O) thrice and then germinated at room temperature (28 ± 2°C; humidity 60 ± 5%) in total darkness for 2-3 days with the help of sterile filter paper. Seedlings were transferred to pots containing autoclaved soil which was composed of coco peat moss (10–15%), coco peat (45–50%), pertile (35–40), NH^+^ (ca.0.09 mg/g), KO (c.0.1 mg/g), zeolite (6-8%) with NO_3_ (ca. 0.205 mg/g), and PO (ca.0.35 mg/g) in a greenhouse at a constant temperature (28 ± 2°C) under a 16/8 hr light and dark period, respectively. Three sets with 5 replicates per set were used for the experiment. Set 1 was kept as control, set 2 contained plants subjected to 1mM Cd stress, and set 3 contained plants subjected to 2mM Cd stress. The concentrations for Cd stress were selected based on the previous report (Khan et al., 2023a) and Cd stress was induced three days after transferring the plantlets into pots by soil irrigation method every three days. For the analyses, leaf samples from each replicate of all the sets were collected, and with the help of liquid nitrogen, the samples were immediately dried and stored at -80°C for RNA extraction.

### RNA extraction, RNA-seq analysis during Cd stress

Leaf samples freeze dried with liquid nitrogen were utilized to extract RNA according to previously reported protocols (Khan et al., 2023a). Briefly, The RNA obtained was diluted until the RNA quantity for each sample reached 100 ng. Three replicates from every treatment were carefully selected and sequenced for subsequent analysis. For RNA-seq analysis, the sequencing platform (Illumina HiSeq2000) was used as per the manufacturer’s procedure, resulting in 51-bp single-end reads. An efficient computational pipeline was used to find differences in gene regulation between the control and plants subjected to Cd stress. The quality was assessed through FastQC. Data was trimmed with the help of TrimGalore. To align and index the data to reference genome, HISAT2 was exploited. Feature Count (subread_v2.0.2) carried out read count quantification, and differential gene analysis was carried out with the help of DESeq2 in the R program. For DEG analysis, p-values were adjusted for multiple testing using the Benjamini-Hochberg procedure to control the false discovery rate (FDR). Genes were considered differentially expressed if they exhibited a log2 fold change (log2FC) greater than ±2, corresponding to a two-fold change in expression.

### Sequences retrieval

The *S. lycopersicum* and *A. thaliana* genes that encodes ATP binding cassettes (ABC) transporters were retrieved from Solgenomics (https://solgenomics.net accessed on 15 July 2023) and TAIR databases (https://www.arabidopsis.org/index.jsp accessed on 15 July 2023). The corresponding genome sequences and protein sequences were downloaded from Solanaceae Genomics Network (SGN, https://solgenomics.net/, accessed on 15 July 2023), and Arabidopsis Information Resource (TAIR, https://www.arabidopsis.org/index.jsp, accessed on 15 July 2023). Consequently, 154 ABC genes from *S. lycopersicum* and 130 ABC genes from Arabidopsis were identified. For valid identification and confirming the presence of the respective domains of the obtained protein sequences, the Simple Modular Architecture Research Tool (SMART, http://smart.embl.de/, accessed on 15 July 2023) was used at E-value <10-5. Only sequences containing the ABC transporter domain were retained, while sequences lacking this domain were excluded from further analysis. This validated dataset was then used for subsequent analysis

### Comparative phylogenetic analysis of ABCs

In order to classify the retrieved genes in *S. lycopersicum* (*SlABCs*), a phylogenetic tree was constructed by employing the Maximum likelihood (ML) technique with the help of software (MEGA 11), keeping the bootstrap replicates (BS) at 1000 times. The procedure above was repeated to construct a combined phylogenetic tree of 154 *SlABCs* with 130 ABCs from *A. thaliana* (*AtABCs*) as well, so as to determine the evolutionary relationship among the genes. The annotations of the resulting phylogenetic trees were performed using Version 1.4.4 of the FigTree (https://mybiosoftware.com/figtree-1-3-1-produce-figures-phylogenetic-trees.html#google_vignette).

### Conserved motif and gene structure analysis in *SlABCs*

Gene Structure Display Server (GSDS), an online tool (https://gsds.gao-lab.org/), was employed to investigate the gene structure, i.e., the number, structure, and organization of exons and introns in the identified *SlABCs*. In order to do so, the coding sequence (CDS) of *SlABCs* was aligned with the genomic sequence. For delving into the details of conserved Motifs in *SlABCs*, another online tool i.e. MEME (Multiple EM for Motif Elicitation) version 5.4.1 (https://meme-suite.org/meme/doc/meme.html) was employed by adjusting the motif number (maximum) to 20, keeping the optimum motif width between 6 to 200 amino acid (aa) residue and fixing the maximum repetitions to “any.”

### Genome-wide synteny/collinearity analysis

The localization and distribution of *SlABCs* on the chromosomes of *S. lycopersicum* was accomplished by genome-wide collinearity analysis using TBtools software. The gene data required for this purpose was downloaded from the NCBI database.

### Calculation of Ks (Synonymous) and Ka (Non-synonymous) values

The selected duplicate gene pairs were subjected to TBtools to compute the Ks and Ka values. The determined values were then used to determine the Ka/Ks ratio. The formula t = Ks/2r was used to calculate the divergence time with r being the representative value (1.5 × 10^-8^) as reported in the literature (Ali et al., 2024).

### Ortholog groups and cis-regulatory modules identification in *SlABCs*

An online tool called OrthoVenn2 (https://orthovenn2.bioinfotoolkits.net/home) was utilized for identification of orthologous groups among the ABC transporter encoding genes of *S. lycopersicum* and *A. thaliana* by analyzing their full-length sequences as reported by (Abiraami et al., 2023). For the mapping and analysis of Cis-regulatory modules, PlantCARE, an online website (http://bioinformatics.psb.ugent.be/webtools/plantcare/html) was explored using 2000 bp sequence upstream of *SlABCs* from *S. lycopersicum* genome with the help of a software (TBtools).

### Predicting network of interaction among proteins

The protein sequences of 154 *SlABCs* were acquired in FASTA format and uploaded online to version 11.5 of STRING’s server (https://string-db.org/) to predict the nature of interaction among different proteins encoded by *SlABCs* and determine the putative function of respective proteins. The network construction was done with the help of selected genes (having significant confidence scores), whereas non-interactive genes were skimmed from the network.

### Prediction of annotated function of *SlABCs*

For the prediction of annotated cellular components, molecular functioning and biological processes involving *SlABCs*, the enrichment analysis of Gene Ontology (GO) categories was performed with the help of online software agriGo (http://bioinfo.cau.edu.cn/agriGO/). Algorithm TopGo ‘elim’ was exploited for the said purpose using the loci of *SlABCs*, which were mined online from the SolGenomics website. Significantly enriched GO Terms (having False Discovery Rate (FDR) and P-value less than 0.05) were considered for the current study, as reported in literature (Khan et al., 2023b).

### qRT-PCR analysis

In order to authenticate the results of the RNA-Seq dataset, 10 genes were selected based on their significant differential expression observed under cadmium (Cd) stress conditions in the RNA-Seq analysis. These genes were chosen to represent the most biologically relevant responses to Cd stress. The selected genes were subjected to qRT-PCR. RNA dilution (0.1µg/µl final concentration) was carried out, followed by the cDNA production with the help of PCR BIOSYSTEMS’s qPCRBIO cDNA Synthesis Kits. qRT-PCR was conducted using BioFACTTM 2× Real-Time PCR Master Mix (Including SYBR^®^ Green I). A total of 20 μl sample containing master mix (10 μl), RNase free water (8 μl), cDNA, and primer (1 μl each) was utilized to carry out PCR reaction with the help of PCR machine (Step One Plus Real-Time PCR System, Life Technologies Holdings Pte Ltd., Singapore). The primers for selected genes were designed with the help of an online tool, i.e., Primer3 (https://bioinfo.ut.ee/primer3-0.4.0/), the details of which are available in (**Supplementary Table S1**). A housekeeping gene, i.e., Actin, was used as an internal control for gene expression normalization. To analyze expression levels of selected genes from control plants and those subjected to Cd stress. Two technical and three biological repeats were employed for each sample in order to minimize experimental error. The conditions for the PCR reaction were as follows: 10 min at 94°C, followed by 35 cycles at 94°C (45 s), 65°C (45 s), and 72°C (1 min), with an extension step at 72°C (10 min). The gene amplification threshold was set at 0.1. Each sample was run three times with three different replicates.

## Results

### Comparative phylogenetic analysis of *SlABCs*

**Figure 1:**
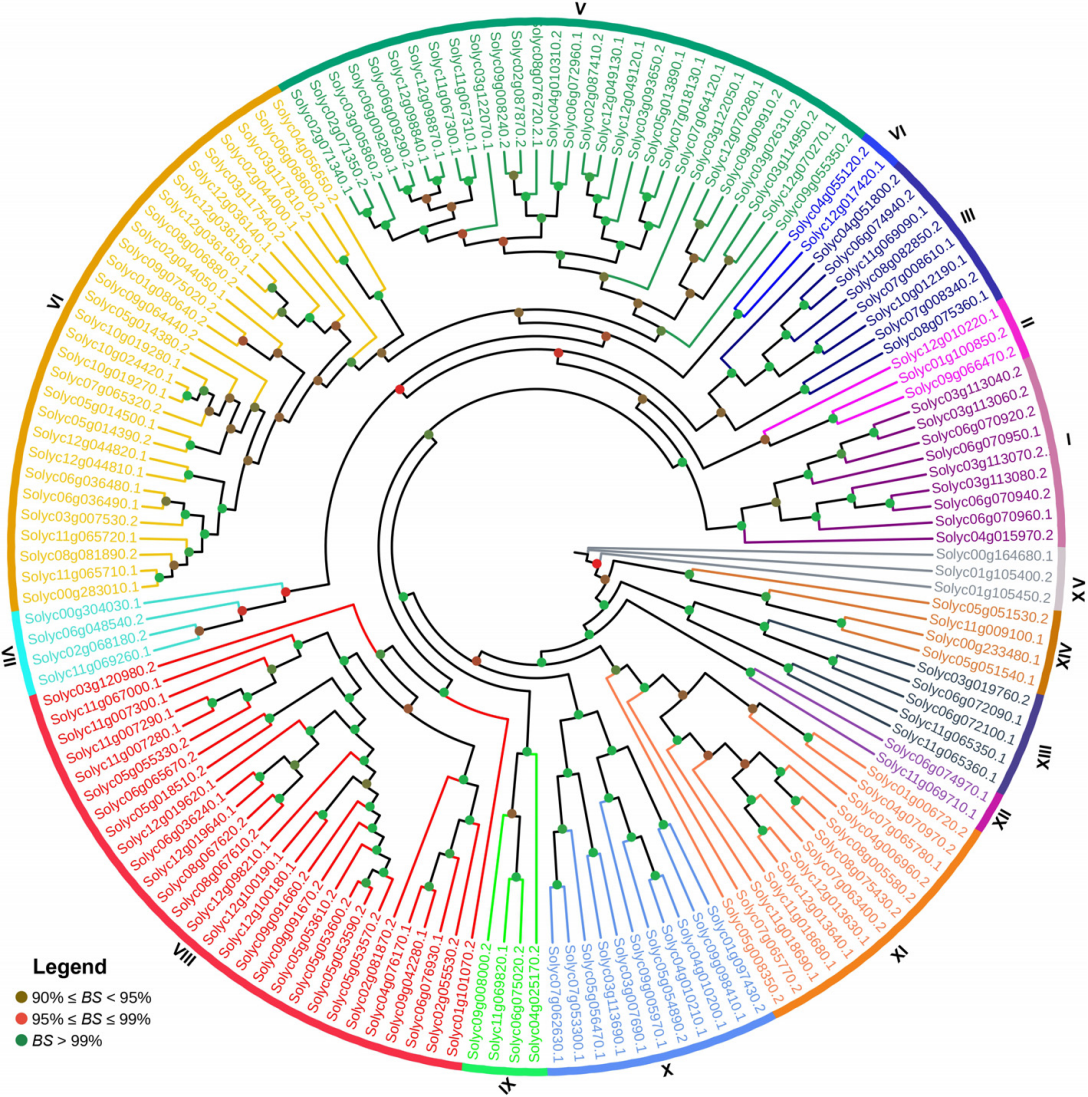
The phylogenetic tree of 154 *SlABCs* constructed using Maximum Likelihood (ML) method with the help of MEGA-11 software with 1000 bootstrap replicates. The major 15 phylogenetic groups are designated as 1-15, respectively.

The phylogenetic tree was constructed using the Maximum Likelihood (ML) method for in-depth analysis of the relationship between the *SlABCs*. As evident in **Figure 1**, the results depicted that the identified *SlABCs* belong to 15 different groups, each labeled and designated by a specified color. More than half of the genes (55.8%) were grouped into 3 out of 15 groups, with Groups 5 and 6 having the maximum number of *SlABCs* (29 each), closely followed by Group 8 (28 *SlABCs*). The rest of the 12 groups collectively had 68 *SlABCs* (44.1%), with Group 11 having 13 *SlABCs*, Group 10 having 11 *SlABCs*, Group 1 having 9 *SlABCs*, Group 3 with 8 *SlABCs*, Group 13 with 5 *SlABCs*, Group 7, Group 9 and Group 14 having 4 *SlABCs* apiece and Group 2 and Group 15 with 3 *SlABCs* each. Groups 2 and 12 had the least *SlABCs*, i.e., 2 *SlABCs* each. A bootstrap value of over 99% in the majority of the clades validates the results above.

**Figure 2:**
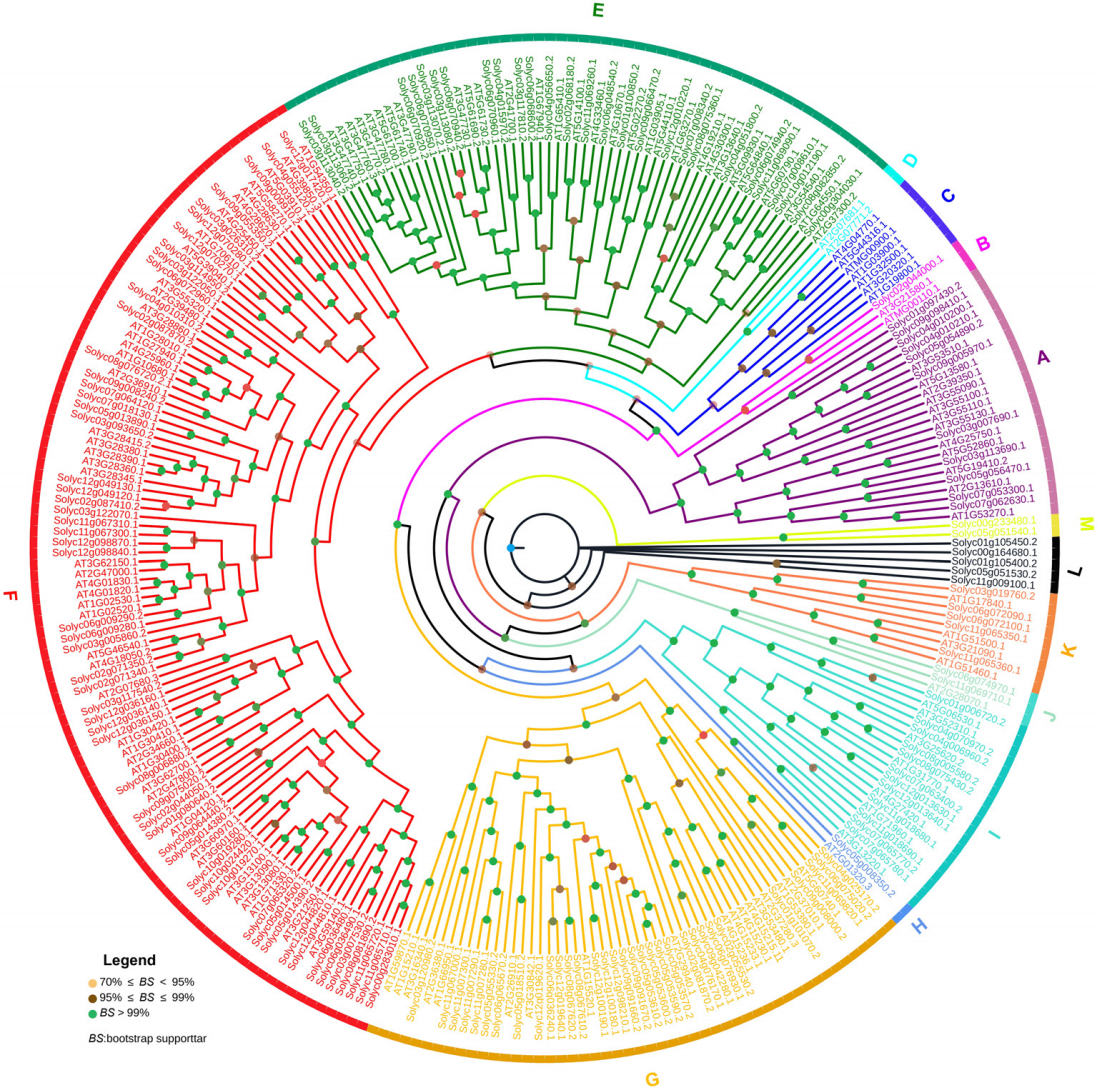
A joint phylogenetic tree was constructed by aligning 154 *SlABCs* and 130 *A. thaliana* ABCs using the Maximum Likelihood (ML) method with the help of MEGA-11 software with 1000 bootstrap replicates. The resulting thirteen groups, labeled A-M respectively, are shown in different colors.

To further understand the evolutionary relationship of the *SlABCs* with *AtABCs*, a combined phylogenetic tree of 154 *SlABCs* and 130 ABC genes belonging to *A. thaliana* (*AtABCs*) was constructed using the aforementioned method. The resulting combined phylogenetic tree, as evident in **Figure 2**, revealed 13 groups named A-M respectively. Apart from Group C and D, which only contained *AtABCs* (7 and 2 respectively), and L and M, which only contained *SlABCs* (5 and 2 respectively), the rest of the 9 groups contained ABC genes from both *S. lycopersicum* and *A. thaliana*. Group F contained the highest number of genes i.e. 102 (46 *AtABCs* and 56 *SlABCs*) followed by Group E with 57 (30 *AtABCs* and 27 *SlABCs*), Group G with 50 (18 *AtABCs* and 32 *SlABCs*), Group A with 23 (12 *AtABCs* and 11 *SlABCs*), Group I with 19 (7 *AtABCs* and 12 *SlABCs*), Group K with 9 (4 *AtABCs* and 5 *SlABCs*), Group B with 3 (2 *AtABCs* and 1 *SlABC*), Group J with 3 (1 *AtABC* and 2 *SlABCs*) and Group H with 2 (1 *AtABC* and 1 *SlABC*). Bootstrap value of over 95%-99% in all the clades validates the abovementioned results.

### Diversity and chromosomal locations of *SlABC* genes

A total of 154 ABC genes were identified in *S. lycopersicum*, which were unequally distributed on chromosomes of *S. lycopersicum* (**Supplementary Table S2**). Maximum number of *SlABCs* were present on chromosome 12 (20 *SlABCs*) followed by chromosome 6 (19 *SlABCs*), chromosome 3 and 11 (17 *SlABCs* each), chromosome 5 (15 *SlABCs*), chromosome 9 (12 *SlABCs*), chromosome 4 (11 *SlABCs*), chromosome 7 (10 *SlABCs* each), chromosome 2 and 8 (9 *SlABCs* each), chromosome 1 (7 *SlABCs*) and chromosome 10 (4 *SlABCs*). The precise location of 4 genes, however, was not identified. Apart from chromosomal distribution, other physical properties of the proteins encoded by *SlABCs*, like isoelectric point, molecular weight, alternative isoforms, number of exons and motifs, etc., also showed diversity (**Supplementary Table S2**). The average pI value was recorded to be 7.64, with *Solyc01g105400.2* showing the lowest pI value (4.31) and *Solyc00g304030.1* displaying the highest value (10.43). Much variation was also noted in encoded proteins and gene length. The number of amino acids ranged from 53 to 1,909 whereas the gene length varied from 244 to 35,740 bp in 154 identified *SlABCs*. The predicted molecular weight ranged between 5.9 kDa and 213.17 kDa; the average molecular weight was 96.85 kDa. Subcellular localization revealed that ABC proteins were located in almost all major organelles, including plastid, vacuole, nucleus mitochondria, endoplasmic reticulum, chloroplast, and Golgi complex (**Supplementary Table S2**).

### Genome-wide synteny/collinearity analysis

**Figure 3:**
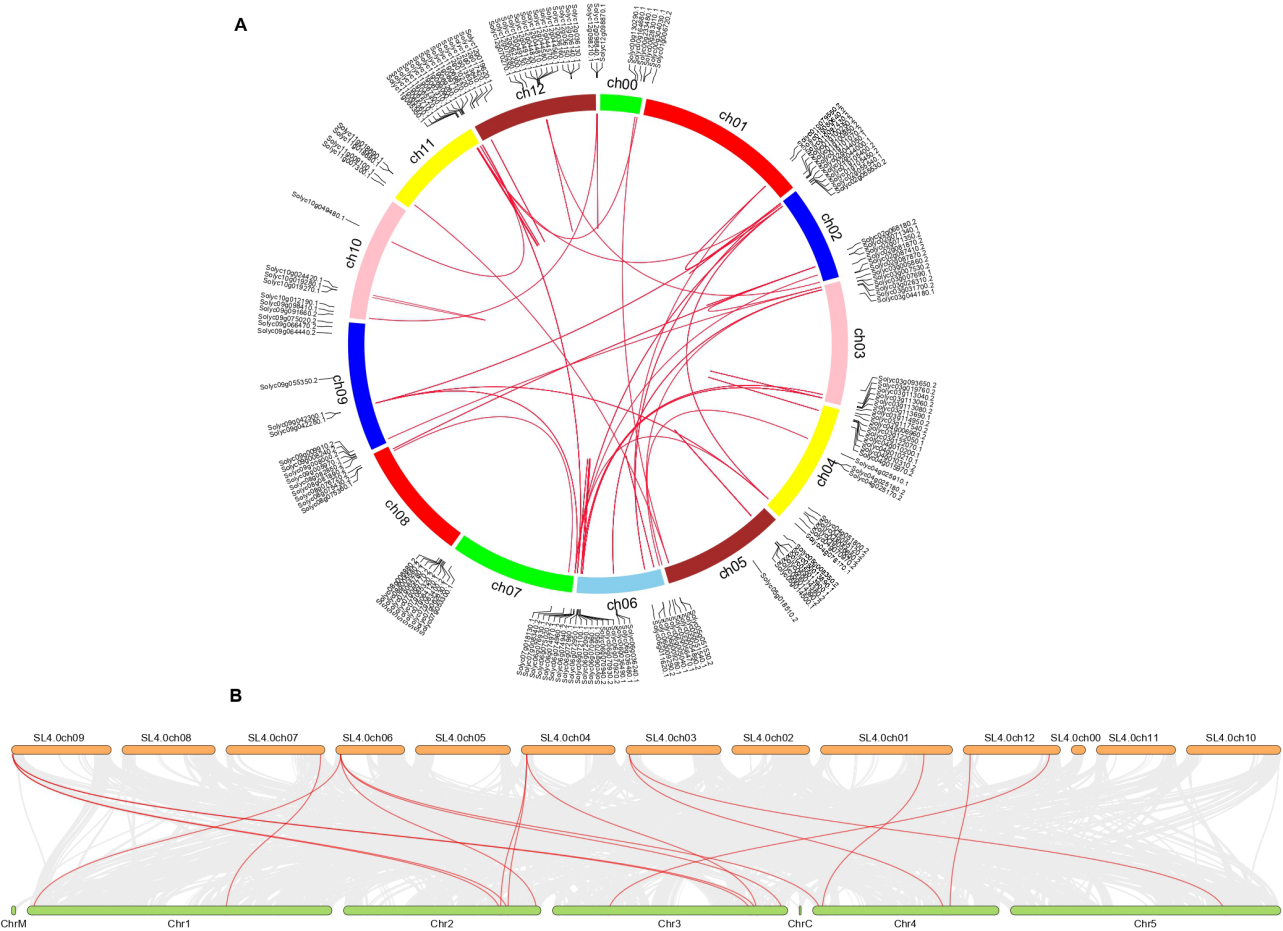
**(A)** Chromosomal positions and inter-chromosomal groups of duplicated *SlABCs* pairs were mapped on chromosomes (Chr1–Chr12) of *S. lycopersicum*. The lines represent the network zone of duplication among *SlABCs*, **(B)** Synteny analysis for identification of locus relationship among *S. lycopersicum* and *A. thaliana* genes.

A collinearity analysis was carried out to analyze the evolutionary relationship and duplication events in *SlABCs*. Duplication of genes is critical in increasing a gene family’s size. A number of *SlABCs* were found to be highly conserved on all the 12 chromosomes of *S. lycopersicum* suggesting multiple duplication events within the *S. lycopersicum* genome. The duplication included both intra-chromosomal and inter-chromosomal mutations (**Figure 3A**). Synteny analysis (**Figure 3B**) for identification of locus relationship among *S. lycopersicum* and *A. thaliana* genes showed the presence of 8 homologous genes. These genes were located on 7 out of the 12 chromosomes of *S. lycopersicum*. Interestingly, the gene on chromosome 6 of *S. lycopersicum* had homologous genes on chromosome 1. 2. 3, and 4 of *A. thaliana*, whereas chromosome 12 had 2 different genes having homologous genes on chromosome 3 and 4 of *A. thaliana*.

In order to estimate the balance between different types of mutations, the Ks and Ka values of selected homologous gene pairs were calculated. The values were used to calculate the ratio between synonymous and non-synonymous values, the mean for which was 0.411. The results showed that the homologous genes evolved through purifying selection, suggesting that mutations that could deteriorate the genes were removed. The whole process was estimated to have occurred approximately 9 to 79 mya depending upon the homologous gene pair in question (**Table 1**).

**Table 1:** Ks/Ka values and divergent time of selected duplicate *SlABCs* pairs.

Seq_1	Seq_2	Ka	Ks	Ka/Ks	Time (MYA)
Solyc00g233480.1	Solyc05g051540.1	0.046258	0.130878	0.353444	9.975479633
Solyc02g081870.2	Solyc06g076930.1	0.127664	0.609289	0.209529	46.43971919
Solyc04g010200.1	Solyc04g010210.1	0.066254	0.229967	0.288101	17.52794222
Solyc04g010210.1	Solyc04g010200.1	0.066254	0.229967	0.288101	17.52794222
Solyc05g051540.1	Solyc00g233480.1	0.120817	0.144999	0.833228	11.05173401
Solyc06g075020.2	Solyc11g069820.1	0.188031	0.552926	0.340066	42.14372784
Solyc11g065350.1	Solyc11g065360.1	0.26768	1.047818	0.255464	79.8641465
Solyc11g065720.1	Solyc11g065710.1	0.181231	0.184919	0.980053	14.09446501
Solyc11g069820.1	Solyc06g075020.2	0.188031	0.552926	0.340066	42.14372784
Solyc12g013630.1	Solyc12g013640.1	0.109691	0.346011	0.317016	26.37277561
Solyc12g013640.1	Solyc12g013630.1	0.109691	0.346011	0.317016	26.37277561

### Conserved motif and gene structure analysis in *SlABCs*

**Figure 4:**
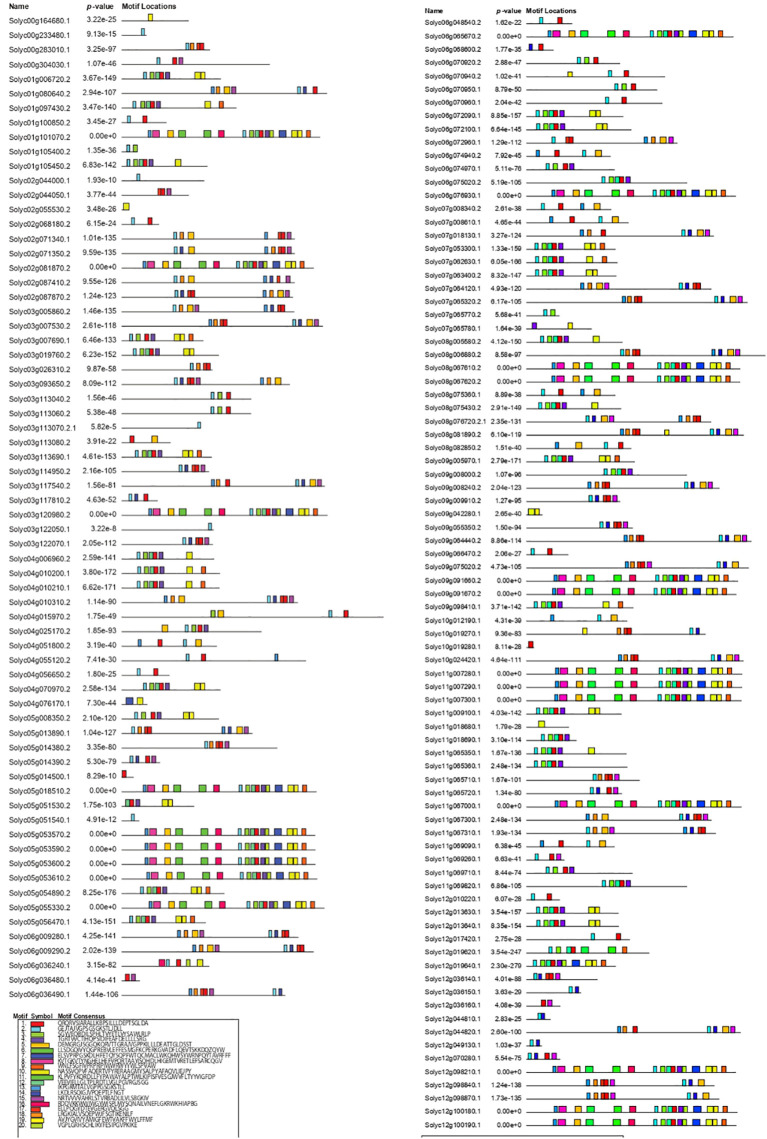
Schematic distribution of 20 conserved motifs in *SlABCs* using MEME Analysis. The motif consensus along with motif symbol is provided in the legend.

**Figure 5:**
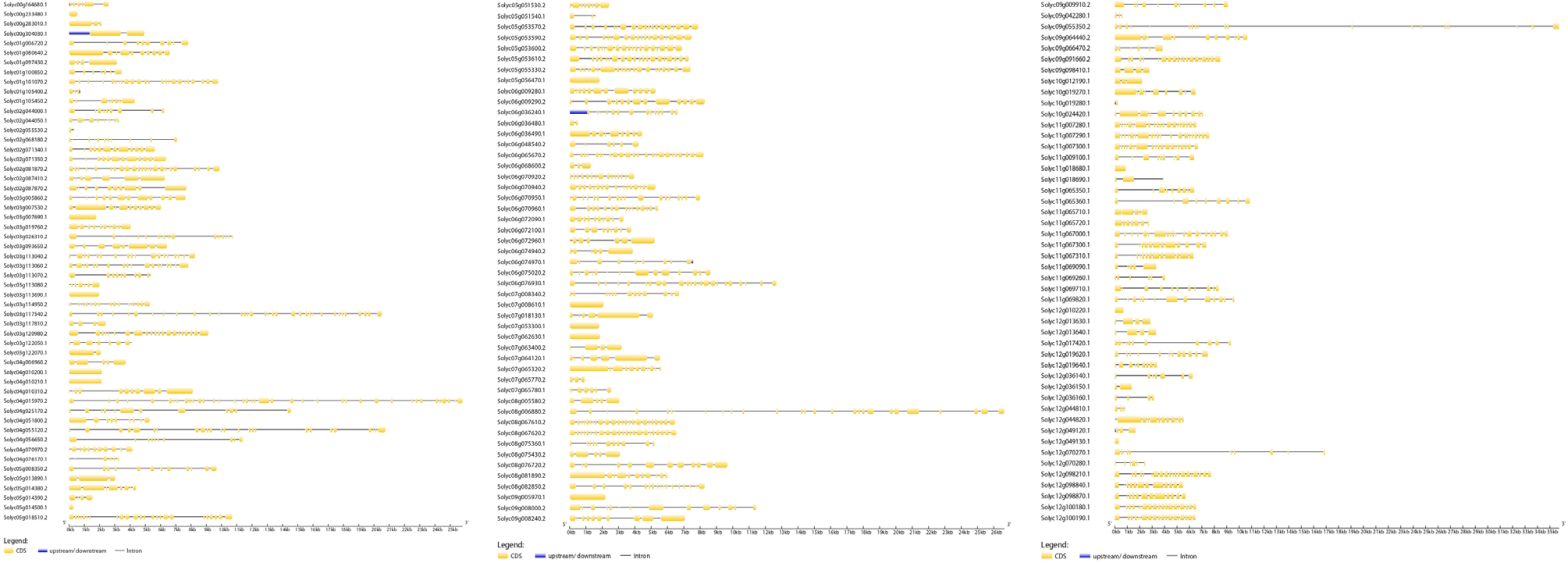
Exon–intron pattern analysis of *SlABCs*. The blue box represents upstream/downstream, the yellow box indicates exons, whereas the lines represent introns.

Examining gene structure and motif composition offers valuable insights into evolutionary history and functional diversity. To explore these aspects, the conserved motifs and exon-intron organization of the 154 *SlABC* genes in *S. lycopersicum* were analyzed (**Figures 4**, **5**). The number of exons in *SlABC* genes ranged from 1 to 40, while introns varied between 0 and 39, indicating considerable structural diversity within this gene family. MEME software identified 20 distinct conserved motifs, reflecting potential functional specialization among *SlABC* genes. Among these motifs, several were identified as Nucleotide Binding Domains (NBDs), crucial for ATP binding and hydrolysis. Specifically, Motif 2 contained the Walker A motif, a key ATP-binding site, while Motif 5 harbored an ABC transporter signature motif essential for transporter function. Additionally, Motif 12 was associated with the Walker B motif, which plays a fundamental role in ATP hydrolysis. Motif 13 was identified as another ATP-binding region commonly found in ABC transporters. The analysis further revealed that full-length ABC transporters predominantly contained two NBDs, whereas half-size ABC transporters had only one. The position of these domains varied across different genes, appearing at either the N-terminal, C-terminal, or in a duplicated arrangement, depending on the specific subfamily.

The conservation of these motifs across multiple *SlABC* genes highlights their strong functional preservation, particularly in ATP-dependent substrate transport. These findings suggest that despite sequence variations, the core functional components of the *SlABC* gene family remain highly conserved.

### Orthologs identification

A total of 42 ortholog clusters (1-42) were identified which are given in **Supplementary Figure S1**. The orange dots represent *A. thaliana* ABC genes (*AtABCs*), whereas the blue dots represent *SlABCs*. The 42 designated clusters show different configurations, including many to many (eight clusters i.e. 1-7 and 10), many to one (six clusters i.e., 8-9, 13-16), one to many (5 clusters i.e. 11-12, 17-19) and one to one (23 clusters, i.e. 20-42) specific ABC members. Interestingly, the orthologs belonging to the same cluster had the same number of motifs, For example, all genes in cluster 2 had the same number of motifs (16) and a similar motif sequence. Similarly, genes belonging to clusters 11 and 12 had seven motifs with a similar motif sequence. Similarly, the number of exons and introns within the same cluster was also similar. For example, all the genes in Cluster 4 had 18 exons and 17 introns, whereas genes in Cluster 5 had a single exon with no introns. The number and sequence of *SlABC* genes in clusters 4 and 5, respectively, are also similar. The same pattern was observed for most of the remaining clusters as well. The above discussion validates the fact that the orthologs have evolved from a common ancestral gene.

According to **Figure 2**, the combined phylogenetic tree divided *SlABCs* and *AtABCs* into 13 groups. Except for Group B, which did not have any orthologs, all the other groups have a number of orthologs i.e. Group F contains 19 orthologs, Group E contained a total of 13 orthologs, Group G have six orthologs, Group A and Group I have five orthologs each, Group K contains two orthologs and Group J and Group H have one ortholog each. Although *A. thaliana* and *S. lycopersicum* belong to different families that diverged millions of years ago, most of the ABCs have orthologous relationships, suggesting that the nature and functions of *AtABCs* and *SlABCs* would show a similar pattern.

### Identification of cis-regulatory modules in *SlABCs*

**Figure 6:**
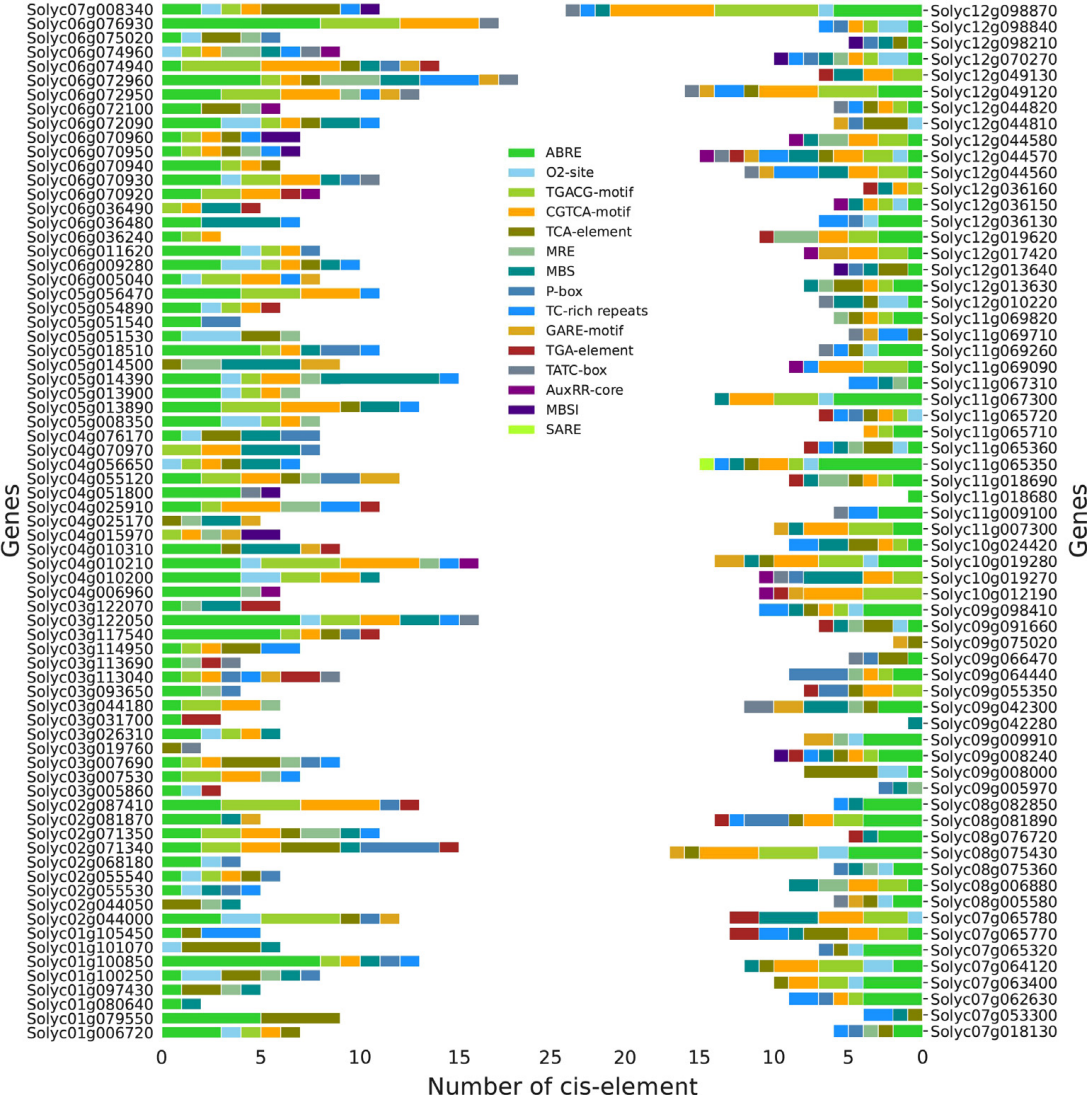
Identified Cis-regulatory modules in *SlABCs*. The legend in the middle shows the designated color for each regulatory module whereas the legend at the bottom displays the number of regulatory elements in individual *SlABCs*.

Various hormones play a critical role in enabling the plant to combat different biotic and abiotic stress conditions. In this regard, 137 *SlABCs* were found to play a role in hormone-responsive cis-regulatory elements (**Figure 6**). A total of 1150 cis-regulatory modules comprising 15 different elements were identified in which ABRE, an ABA-responsive element, was the most repeated cis-regulatory element present (279) found in 114 *SlABCs* followed by CGTCA-motif (155), a Methyl jasmonate (MeJA) responsive element and TGACG-motif (155) which is involved in the regulation of salicylic acid (SA) and MeJA signaling pathways which were identified in 54 and 53 *SlABCs* respectively. The number of cis-regulatory modules found in individual *SlABCs* showed tremendous diversity and ranged between 1-24 with *Solyc12g098870.1* having the most number of cis-regulatory modules (7 TGACG-motifs and CGTCA-motifs, 6 ABRE and 1 O_2_-site, TC-rich repeats and TATC-box) and two *SlABCs* (*Solyc11g018680.1* and *Solyc09g042280.1*) having only one cis-regulatory module. Interestingly, only one *SlABC* (*Solyc11g065350.1*) contained the SARE motif, a SA-responsive element. From the above discussion, it is evident that all of the *SlABCs* contain at least one stress-responsive cis-regulatory element, suggesting their role in combating a variety of biotic and abiotic stress to which plant is constantly exposed due to their sessile nature.

### Protein-protein interaction based functional annotation of *SlABCs*

**Figure 7:**
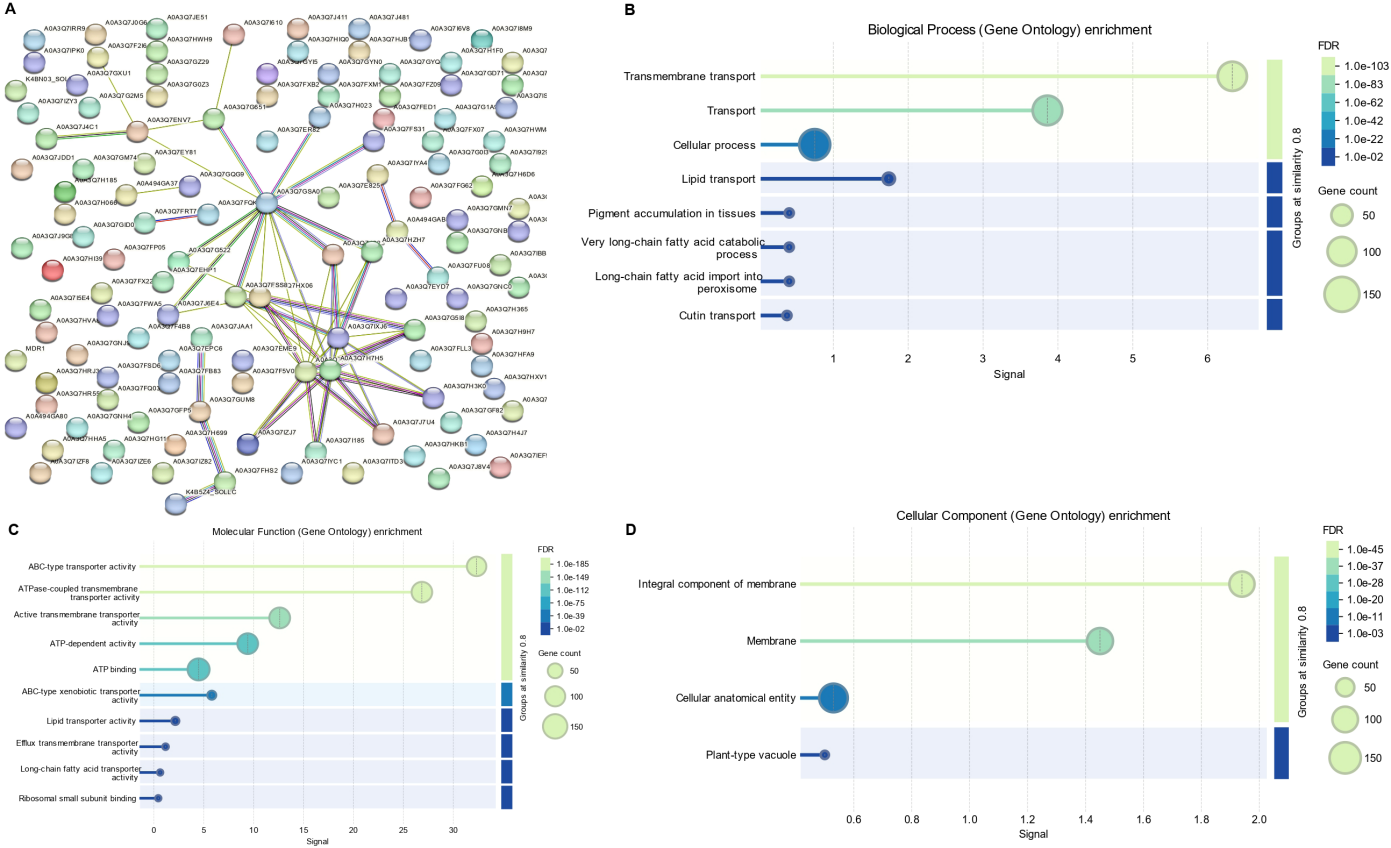
**(A)** Network of Protein-protein interaction among *SlABCs* based on their available information. The prediction of interacting network was carried out using STRING analysis. Different line colors represent the types of evidence for the associations **(B)** Gene ontology (GO) enrichment of *SlABCs* representing biological process **(C)** Gene ontology (GO) enrichment of *SlABCs* representing molecular functions **(D)** Gene ontology (GO) enrichment of *SlABCs* representing cellular components. The sizes of the circles indicate the number of genes in each category, while the x-axis bars indicate fold enrichment.

Protein-protein interactions (PPI) offer valuable insights into the biological functions of proteins, as those involved in similar pathways often interact with each other. To better understand the functional associations of SlABC transporters, a protein-protein interaction (PPI) network was constructed using STRING (**Figure 7A**). The analysis revealed a well-connected network, where several *SlABC* transporters formed central hubs, suggesting their significant role in intracellular transport and regulatory processes. The functional enrichment of interacting proteins highlighted key biological processes, including ATP-dependent transport, xenobiotic efflux, lipid transport, and long-chain fatty acid metabolism. Notably, the presence of ABC-type xenobiotic transporters suggests that some *SlABC* proteins may be involved in detoxification, while interactions with lipid transporters indicate a potential role in maintaining membrane stability and homeostasis. Gene ontology (GO) enrichment further confirmed associations with transmembrane transport, ATP binding, and efflux mechanisms, emphasizing their importance in energy-dependent substrate transport.

These findings prove that *SlABC* transporters are functionally diverse and play crucial roles in cellular transport, stress adaptation, and metabolic processes in *S. lycopersicum*. The PPI network suggests that certain SlABC transporters act as key regulators of nutrient transport and environmental stress responses, potentially contributing to overall plant resilience and development.

### Prediction of the annotated function of *SlABCs* using GO analysis

To predict the annotated function of the identified *SlABCs*, Gene Ontology (GO) enrichment analysis was carried out through online GO tools. As evident in **Figure 7**, the *SlABCs* were involved in a number of biological (**Figure 7B**), molecular (**Figure 7C**), and cellular components (**Figure 7D**). Key biological processes include transport (GO:0006810), localization (GO:0051179), the establishment of localization (GO:0051234), and transmembrane transport (GO:0055085), whereas molecular functions included a wide range of activities like active transmembrane transporter activity (GO:0022804), ATP binding (GO:0005524), adenyl ribonucleotide binding (GO:0032559), purine ribonucleoside triphosphate binding (GO:0035639), transmembrane transporter activity (GO:0022857), ribonucleotide binding (GO:0032553), carbohydrate derivative binding (GO:0097367), anion binding (GO:0043168) and ABC-type transporter activity (GO:0140359) etc.

### Gene expression analysis of *SlABCs* under Cd stress

**Figure 8:**
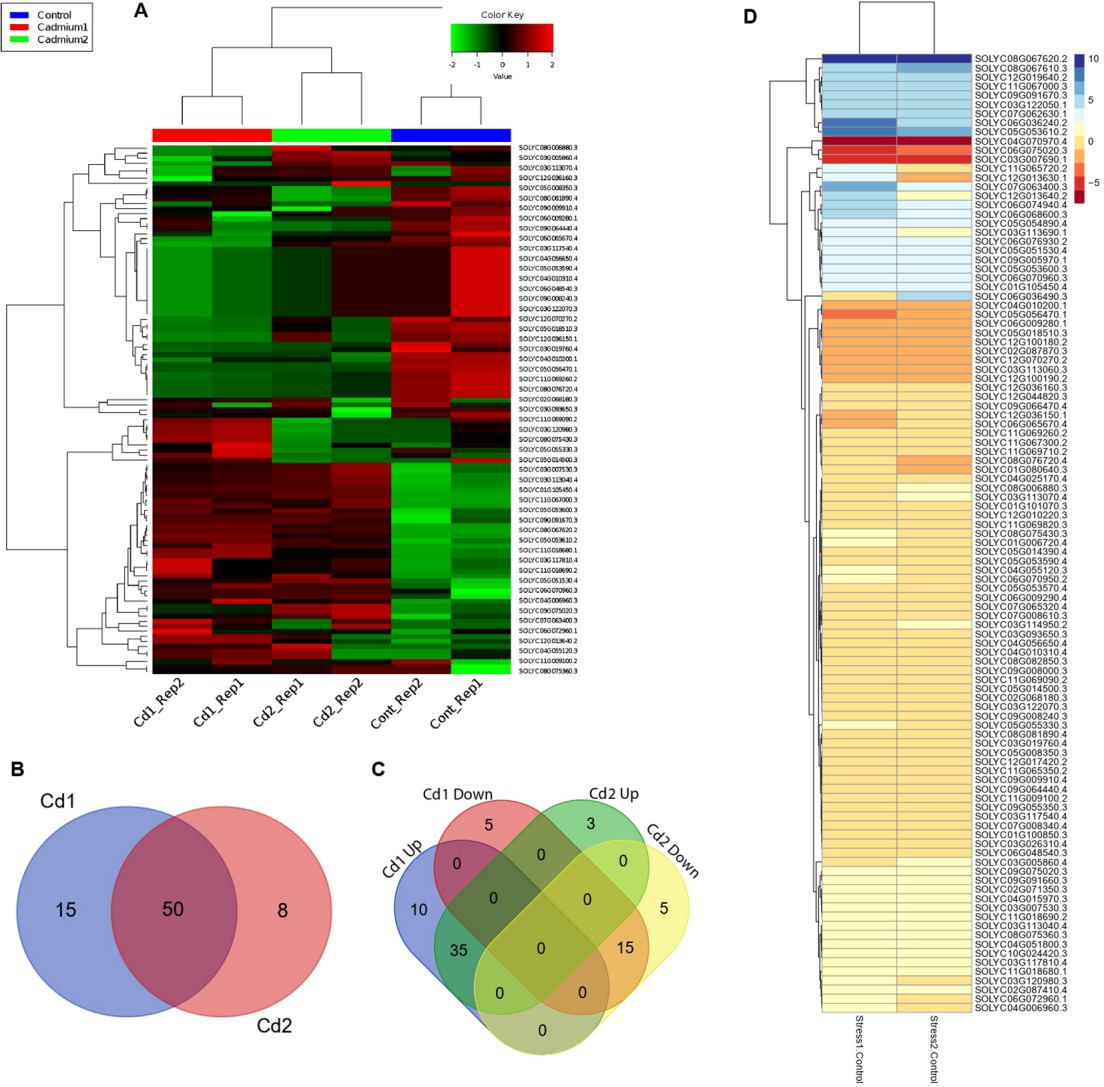
**(A)** Hierarchical clustering indicate the substantial difference in *SlABCs* genes induced by 1 mM and 2 mM Cd stress **(B)** Venn Diagram showing the number of *SlABCs* that were upregulated in response to Cd1 (1mM Cd) and Cd2 (2mM Cd) **(C)** Venn diagram showing the number of differentially expressed *SlABC* gene in plants exposed to Cd1 (1mM Cd) and Cd2 (2mM Cd) **(D)** Heatmap demonstrating the differentially expressed gene pattern. Expression changes in the genes are represented by the color scheme displayed in the legend.

Plants in their natural habitat had to face a number of extreme conditions like drought, salinity, and heavy metal-induced stress. A variety of ABC isoforms are well known for their role in the reclamation of heavy metal-induced stress; however, the response of *SlABCs* to Cd-induced stress has not been explored to the best of our knowledge. In order to address the existing research gap, the *S. lycopersicum* plantlets were exposed to two different concentrations of Cd (1 mM and 2 mM) and expression levels of targeted *SlABCs* was evaluated using RNA-seq analysis (**Figure 8**). About 105 selected *SlABCs* were found to be expressed during Cd stress, with several genes showing differential expression patterns. 15 *SlABCs* were expressed in response to 1mM Cd stress alone, 8 *SlABCs* were expressed in response to 2 mM Cd, and 50 *SlABCs* showed expression in both 1mM and 2mM Cd stress (**Figure 8B**). In the case of 1mM Cd stress, 10 *SlABCs* were upregulated, whereas 5 *SlABCs* were downregulated. Similarly, 3 *SlABCs* were upregulated, and 5 *SlABCs* were downregulated in response to 2mM Cd stress, whereas 35 *SlABCs* were upregulated and 15 *SlABCs* were downregulated in response to both 1mM and 2mM Cd stress (**Figure 8C**).

### qRT-PCR based validation of gene expression

**Figure 9:**
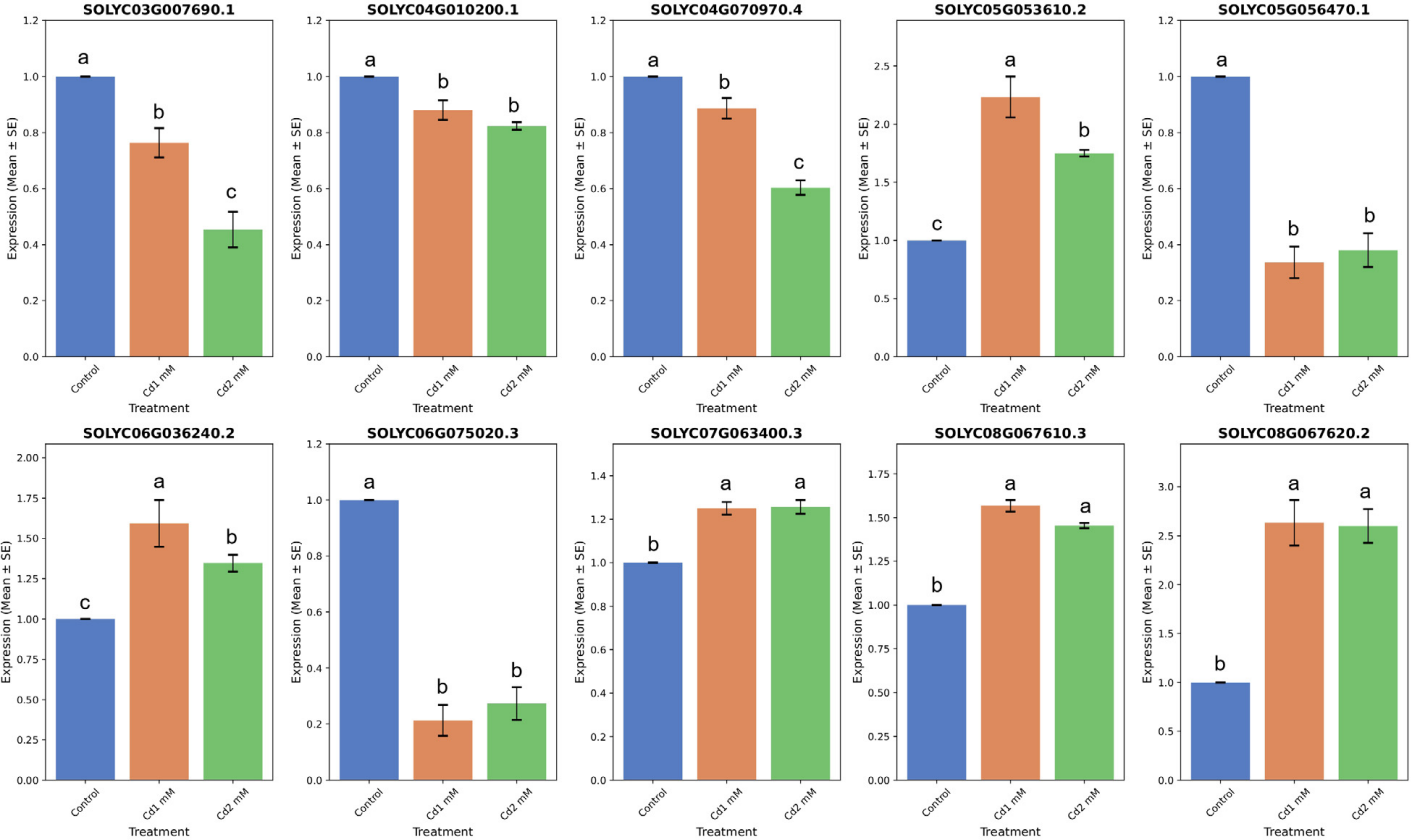
qRT-PCR based quantification of selected *SlABCs* for the validation of RT-PCR results. The x-axis displays the type of treatment, whereas the y-axis displays the gene expression level. The data is expressed in terms of Mean ± SE. Different letters over bar indicates significant difference in treatment according to Duncan's multiple range test DMRT p≤ 0.05).

Using specific primers, the RNA-seq-based gene expression data was validated by subjecting 10 *SlABCs*, i.e., *Solyc03g007690.1*, *Solyc04g010200.1*, *Solyc04g070970.4*, *Solyc05g053610.2*, *Solyc05g056470.1*, *Solyc06g036240.2*, *Solyc06g075020.3*, *Solyc07g063400.3*, *Solyc08g067610.3*, and *Solyc08g067620.2* to qRT-PCR for analyzing the relative gene expression levels. There was a strong correlation between gene expression results obtained by both RT-PCR and qRT-PCR. For instance, 5 *SlABCs* i.e. *Solyc03g007690.1*, *Solyc04g010200.1*, *Solyc04g070970.4*, *Solyc05g056470.1* and *Solyc06g075020.3* were downregulated in both RNA-seq (**Figure 8D**) and qRT-PCR (**Figure 9**) data whereas the other 5 *SlABCs* i.e. *Solyc05g053610.2*, *Solyc06g036240.2*, *Solyc07g063400.3*, *Solyc08g067610.3*, and *Solyc08g067620.2* were all upregulated in both RNA-seq and qRT-PCR based gene expression analysis thereby validating the accuracy our results.

## Discussion

Plants are among organisms that are extremely sensitive to any change in the environment. Change in temperature, humidity, salinity, rainfall patterns radiation etc., negatively impacts the growth of plants (Georgieva and Vassileva, 2023). This incurs a number of problems as plants are exposed to a number of biotic and abiotic stress conditions throughout their life (Nawaz et al., 2023). Due to the sessile nature of plants, abiotic stress factors like extreme temperature, salinity, drought, nutrient deficiency, floods, and elevated levels of HMs are detrimental to plants’ growth, survival, and overall yields (Zhang et al., 2023). Plants respond to abiotic stress by involving a number of complex biochemical pathways controlled by a plethora of genes (Javaid et al., 2022). Among them, ABCs are well known for their significant role in the management of abiotic stress (Dahuja et al., 2021). In a detailed study on *Pyrus bretchneideri*, 82 ABC genes were found to be involved in salt stress, and 91 genes were identified as drought-responsive genes, proving the significant role of ABC genes in response to abiotic stress (Kou et al., 2024). Yu et al. reported the upregulation of ABCB genes in response to different abiotic stresses like drought, salinity, and cold (Yu et al., 2017). Another study revealed the regulation of 51 ABCs upon exposure to abiotic stress (Nguyen et al., 2014). In *Glycyrrhiza glabra*, 7 out of 9 ABCs studied showed upregulation in response to drought stress (Devi et al., 2024) suggesting active participation of ABC genes in abiotic stress conditions. ABCs have also been reported for their role in heavy metal tolerance. Guo et al. reported the regulation of ABCs in response to different concentrations of lead (Guo et al., 2022). Arsenic toxicity in rice was reported to be mitigated by the active transport of arsenic to the vacuole for detoxification by ABCs (Rekha et al., 2021). In rice, 47 ABCs were upregulated in response to HMs (Nguyen et al., 2014).

Although not much work has been done on the role of ABCs in mitigating abiotic stress in *S. lycopersicum*, the above literature strongly suggests that they play a vital role in managing abiotic stress in plants. The current study, therefore, explored the role of ABC genes in promoting heavy metal tolerance in *S. lycopersicum*. A total of 154 *SlABCs* were identified which belong to 15 different groups based on phylogenetic analysis. The identified *SlABCs* were distributed across all 12 chromosomes of *S. lycopersicum*. Within the cell, these *SlABCs* were found to be distributed across all the major organelles of the cells like vacuole, nucleus, mitochondria, endoplasmic reticulum and golgi complexes. Both Golgi bodies and the endoplasmic reticulum plays a vital roles in the transportation of materials within the cells (Mohan et al., 2023). One of the main mechanism involved in HMs tolerance is its sequestration into vacuole to avoid oxidative damage caused by HMs (Zhang et al., 2018). The presence of *SlABCs* in all of these vital organelles may therefore enable the movement of Cd with the help of various cellular organelles into the vacuole.

Our study revealed the presence of 1150 hormone-responsive elements in *SlABCs*, including ABA, SA, and MeJA-responsive elements, all of which have been reported for their active participation in the regulation of heavy metal stress (Pompeu et al., 2017; Guo et al., 2018; Lu et al., 2020; Gupta and Seth, 2021; Kamran et al., 2021; Wei et al., 2021; Fan et al., 2022; Wei et al., 2022; Sun et al., 2023; Wu et al., 2023; Qin et al., 2024) suggesting that *SlABCs* did play critical role in Cd tolerance by hormonal transport. In a major study, transcriptome analysis of 29,921 differentially expressed genes revealed that the majority of these genes were responsible for signal transduction and transport of plant hormones and antioxidant processes (Li et al., 2023) which further signifies the importance of these hormones in combating HMs stress. GO analysis of the SlABCs revealed their involvement in a number of biological, molecular, and cellular functions, including the establishment of localization, ATP binding activity, transmembrane transport, and ABC-type transporter activity. Previous studies on soybean also revealed active involvement of ABC transporters in establishment of localization, ATPase activity, cellular transport and nucleotide binding (Naaz et al., 2023). The protein-protein interaction analysis (**Figure 7A**) further supports the functional significance of *SlABC* transporters, revealing their involvement in ATP-driven transport, xenobiotic detoxification, and lipid metabolism. The identification of central hub proteins suggests that some *SlABC* genes play key regulatory roles, particularly in stress adaptation and intracellular transport processes in *S. lycopersicum*.

Differential expression of ABCs in response to Cd in many plants has been reported previously. In rice, a G-type ABC transporter *OsABCG36* was upregulated in root samples exposed to Cd. When *OsABCG36* was knocked out, the plants demonstrated higher sensitivity to Cd stress (Fu et al., 2019). Similar results were reported in another study conducted on *A. thaliana*. The *ABCG36* gene from poplar (*PtoABCG36*) was transformed into *A. thaliana*. The resulting transgenic *A. thaliana* resulted in improved Cd accumulation and resistance to Cd stress (Wang et al., 2019). Naaz et al (Naaz et al., 2023), explored potential role of ABCs from soybean (GmABCs) in mitigation of Cd stress. Relative expression analysis of 10 GmABCs revealed up to 50-folds greater expression in plants exposed to Cd as compared to control. Out of these, 7 GmABCs were observed to be upregulated and 3 GmABCs were downregulated upon exposure to Cd. In an experiment on *A. thaliana*, an *AtABCC3* defective and overexpressed variety were subjected to different Cd concentrations. Whereas *AtABCC3* defective variety showed increased sensitivity to Cd even at low concentrations, the *AtABCC3* overexpression resulted in Cd sequestration into vacuole offering Cd tolerance (Brunetti et al., 2015). Similar results were reported for *AtABCC1 and AtABCC2* defective varieties (Park et al., 2012), which signifies the role of ABCs in conferring Cd tolerance.

In the current study, RNA-seq data analysis revealed the regulation of a number of *SlABCs* in response to Cd stress. Both 1 mM and 2mM Cd concentration resulted in regulation of *SlABCs* which indicates its potential involvement in Cd stress. About 10 highly expressed *SlABCs* were subjected to qRT-PCR, which showed expression patterns similar to those in RNA-seq, which validates the accuracy of our results. Bovet et al (Bovet et al., 2005). explored ABC genes in *A. thaliana* for their possible role in Cd tolerance. A number of ABCs were reportedly expressed in plants subjected to Cd stress, highlighting the possible role of *AtABCs* in Cd stress. Our study revealed a number of orthologs between *S. lycopersicum* and *A. thaliana* (**Supplementary Figure S1**), validating the role of *SlABCs* in Cd stress. Expression data in our study revealed *Solyc08g006880.3* upregulation in response to Cd stress, which is an ortholog of *AT1G30400.1* and *AT2G34660.1* (**Figure 2**; **Supplementary Figure S1**). Both these *AtABCs* are known to be involved in the detoxification of Cd (Park et al., 2012). Upregulation of *Solyc03g120980.3* in plants exposed to Cd was also observed, which is in the same phylogenetic clade (**Figure 2**) as *AT1G59870.1*, a gene previously reported for its role in Cd tolerance in *A. thaliana* (Kim et al., 2007). *Solyc12g044820.3*, expressed in response to Cd stress, shares the same clade with *AT3G21250.4*, previously reported in Cd tolerance in *A. thaliana* (Gaillard et al., 2008). *Solyc05g014390.2* and *Solyc07g065320.2* were also expressed in response to different concentrations of Cd. Both these *SlABCs* are present in the same clade as *AT3G13080.1* (**Figure 2**), which has been previously reported for its active involvement in the transport of and detoxification of Cd (Bovet et al., 2005), thus validating our results.

Despite useful insights on the role of ABC transporter proteins in mitigating Cd stress in *S. lycopersicum*, there are certain limitations. The experimental analysis was conducted in a controlled environment in growth room with specific conditions however, in the natural environment, plants have to cope with variety of adverse conditions like temperature variation, scarcity or abundance of water, salinity, radiations and availability of nutrients (Mareri et al., 2022). Such adverse conditions, combined with Cd stress may affect the differential expression of *SlABCs*. These factors also need to be brought under consideration in future. For comprehensive understanding of complex physiological, biochemical and genetic pathways involved in mitigation of Cd stress in *S. lycopersicum*, further research exploring influence of epigenetic changes is of paramount importance. With comprehensive knowledge of how the differential gene expression may be effected in the natural environment, tailor-made plant breeding strategies could be employed for production of cultivars with high yields despite unfavorable conditions.

## Conclusion

This study identified 154 *S. lycopersicum* ABC transporter genes (*SlABCs*), categorized into 15 groups, with evolutionary conservation and functional diversity. Chromosomal mapping revealed an uneven distribution, with the highest density on chromosome 12. Gene structure and motif analyses highlighted their complexity and diverse functions. Synteny and collinearity analyses demonstrated that duplication events and purifying selection significantly shaped this gene family. Cis-regulatory element analysis linked *SlABCs* to stress responses, particularly ABA, MeJA, and SA pathways. RNA-Seq data revealed differential expressions of several *SlABCs* under Cd stress, with specific genes showing significant upregulation. qRT-PCR further validated these findings, confirming their involvement in Cd detoxification. With these findings, further research into genome editing targeting the highly expressed *SlABCs* could be employed for production of varieties with superior yields. Gene Ontology enrichment and protein interaction analyses supported their roles in transmembrane transport and cellular homeostasis. Overall, *SlABCs* are critical in stress adaptation, particularly in heavy metal detoxification, offering potential targets for genetic and biotechnological interventions to enhance stress resilience in *S. lycopersicum* and other crops. Insights from the current study could prove useful for plant breeders to develop cultivars that could withstand high levels of HMs including Cd.

## Data Availability

The data presented in the study are deposited in the National Center for Biotechnology Information (NCBI) repository, accession number PRJNA913645.

## Supplementary material

Supplementary Table 1 Details of Primers designed for selected genes

Supplementary Table 2 Different characteristic features of *SlABCs*.

Supplementary Figure 1 Ortholog clusters outlining the *AtABCs* (orange) and *SlABCs* (blue). Different clusters are labelled as 1-42 respectively.
