# Supplementary figures and images for: Exploring the role of ATP-binding cassette transporters in tomato (Solanum lycopersicum) under cadmium stress through genome-wide and transcriptomic analysis
Source: Front Plant Sci. 2025 Mar 18;16:1536178. doi: 10.3389/fpls.2025.1536178 (PMC11958947; doi:10.3389/fpls.2025.1536178)

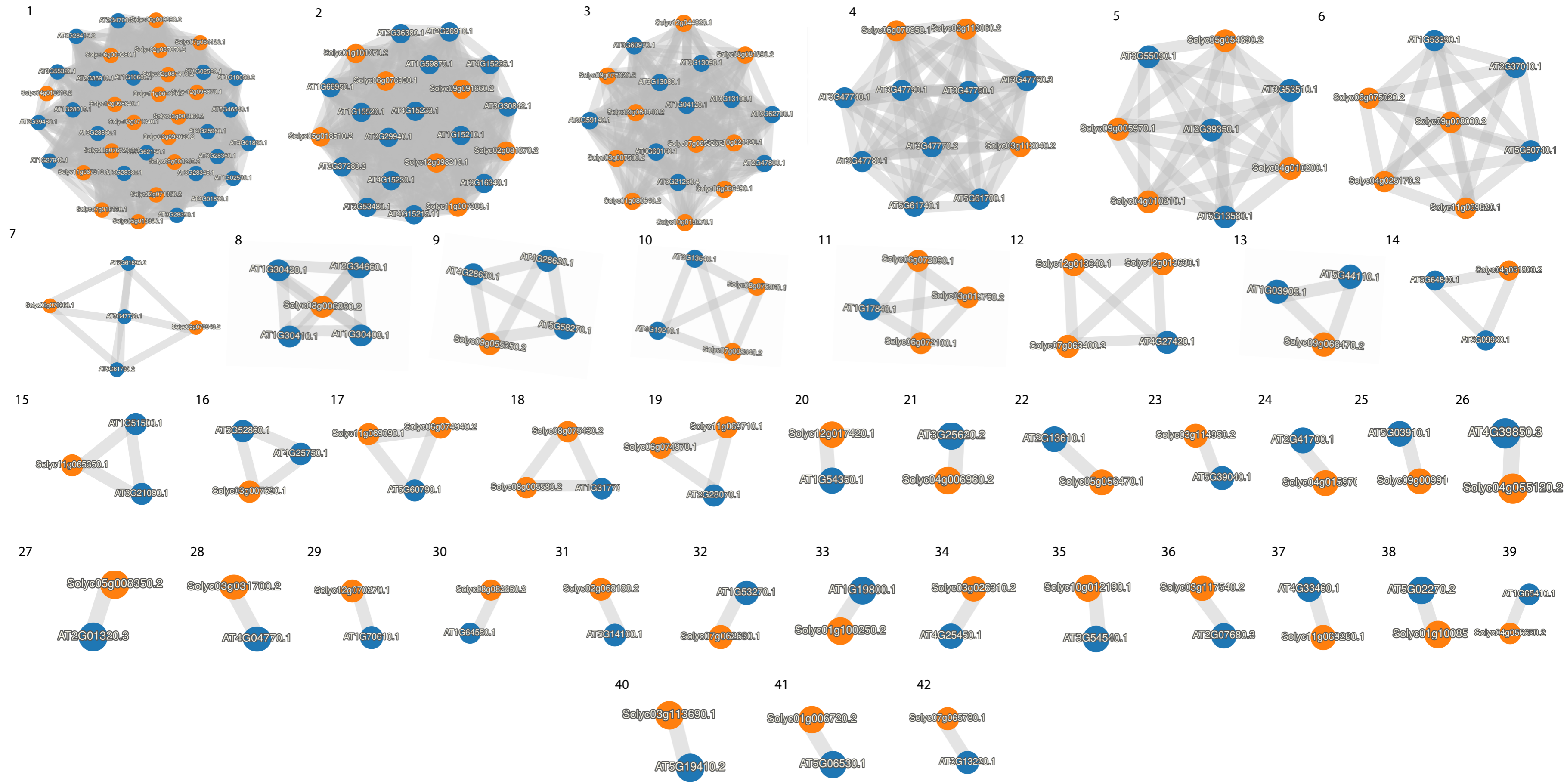

Supplement: Supplementary Figure 1 — Ortholog clusters outlining the AtABCs (orange) and SlABCs (blue). Different clusters are labelled as 1-42 respectively. [file DataSheet1.pdf]
